# Data-Driven Bias Correction and Defect Diagnosis Model for In-Service Vehicle Acceleration Measurements

**DOI:** 10.3390/s20030872

**Published:** 2020-02-06

**Authors:** Lei Bai, Rengkui Liu, Qing Li

**Affiliations:** 1 State Key Laboratory of Rail Traffic Control and Safety; School of Traffic and Transportation, Beijing Jiaotong University, Beijing 100044, China; 2 Beijing JRM Track Technology Service Co., Ltd., Beijing 100070, China; blbailei@gmail.com; 3 Beijing National Railway Research & Design Institute of Signal & Communication Group Co., Ltd., Beijing 100070, China; 13114231@bjtu.edu.cn

**Keywords:** railway track, in-service vehicle acceleration measurement, bias correction, defect diagnosis, data-driven model

## Abstract

Track quality instruments use low-cost accelerometers placed on or attached to the floors of operating trains, and these instruments collect substantial amounts of data over short inspection periods. The measurements collected by the instruments are the main data source for track irregularity evaluation. However, considerable measurement bias exists in the vertical and lateral vibration data obtained from such instruments. False positive track vibration defects detected by track quality instruments occur frequently. This results in considerable time and effort being expended needlessly because maintenance workers have to visit the railway track sites to check and review the track vibration defects. Therefore, we propose a model for data-driven bias correction and defect diagnosis for in-service vehicle acceleration measurements based on track degradation characteristics. Substantial amounts of historical track measurement data from different inspection methods were mined extensively to eliminate the false positive detection of track vibration defects and diagnose the causes of track vibration defects. Actual measurement data from the Lanxin Railway were used to validate our proposed model. The success rate achieved in identifying false positive track vibration defects was 84.1%, and that in track vibration defect diagnosis was 75.8%. These high success rates suggest that the proposed model can be of practical use in improving railway track maintenance management.

## 1. Introduction

**Figure 1:**
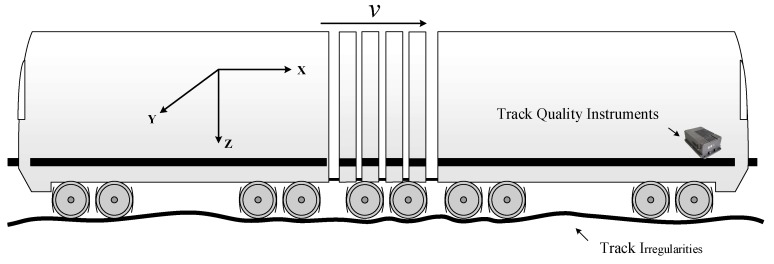
Track geometry measurement using track quality instruments.

Track health depends on the track geometry condition and the condition of each track component (such as rails, sleepers, fasteners, and ballast) [1]. Total track inspection activities therefore consist of track geometry inspection and track component inspection. Total track inspections are classified as on-track inspections (such as track walking and crossing inspection) and on-board inspections (such as track geometry cars and laser clearance reviews) [2,3,4]. Track geometry cars are perceived as crucial inspection devices to detect track irregularities. However, as they are expensive to use, their use frequency is typically limited. For example, in the railway management system of China, one railway line can only be inspected twice per month using a track geometry car [5]. As an alternative, track quality instruments are used frequently. These are low-cost accelerometers placed on or attached to the cabin floors of in-service trains [6]. In-service train vehicles and railway tracks comprise coupled vibration systems. Track irregularities excite dynamic responses from train vehicles. Vertical and lateral vibration data from accelerometers in the cabin of a train can reflect track irregularities. A track quality instrument uses inner accelerometers to measure the vibrations of an operating train, as shown in Figure 1. Cabin acceleration signals in the lateral and vertical directions are obtained to detect track irregularities. Track quality instruments are classified as portable instruments or cabin-mounted instruments. Railway staff periodically place portable track quality instruments on floors of train cabins. A cabin-mounted track quality instrument can be protected from the external environment, as it is attached to the floor of a train cabin.

The inspection frequency achievable with track quality instruments is multiple times per day. This high inspection frequency achievable with track quality instruments enables inexpensive and near “real-time” track monitoring, and can provide near “real-time” track condition information to railway managers. This means that severe track defects can be detected sooner and that a substantial amount of vibration data can be collected from operating trains in regular service. Vibration data constitute a substantial proportion of all track measurement data collected by the different inspection methods and are the main type of data used in assessing track irregularity conditions [1].

When the measured vibrations of a train cabin exceed a specified threshold, the corresponding cabin vibration is termed a “track vibration defect.” In China’s railway management system, when there are severe track vibration defects in railway lines, maintenance workers are usually assigned to visit the site to check and review these defects using other inspection tools, such as track geometry trolleys [5]. Track geometry trolleys are portable and are pushed manually. False positive detections of track vibration defects by track quality instruments are identified by these workers and the locations of actual track vibration defects are determined accurately. The causes of actual track vibration defects (i.e., severe vertical and lateral vibration of the cabin) are also diagnosed by these workers.

Owing to the influence of the rolling stock itself and the influence of the external environment, significant measurement biases occur in the vibration data obtained from track quality instruments. False positive detections of track vibration defects by track quality instruments occur frequently. This causes considerable problems for the maintenance workers at the railway site, as considerable time and effort are required to check and review track vibration defects. Whether the China Railway Corporation should continue to use track quality instruments to assess track conditions is a dilemma. Managers need to use vibration measurements to monitor track conditions. However, this increases the workload of maintenance workers and may lead to reduced track maintenance window utilization and increased maintenance costs.

Detecting the railway track geometry condition from an in-service vehicle is an attractive challenge [7]. A significant amount of data can be obtained from track quality instruments placed/mounted on an in-service vehicle. However, little research has been conducted on how to address the false positive detections of track vibration defects by such instruments. Many researchers have proposed models for detecting track defects using in-service vehicle acceleration measurements [8,9,10,11,12,13,14,15,16,17,18,19,20,21,22]. Most of the models or algorithms proposed are based solely on vehicle acceleration measurements. Multiple condition measurements obtained using different inspection methods are seldom combined. Accordingly, this study was conducted to fill the research gap.

Lederman et al. [8] proposed a data-driven approach that extracts features to identify two types of track changes (specifically, track replacement and tamping) by using vertical and lateral vibration data from accelerometers in the cabin of an in-service train. OBrien et al. [9,10,11,12] proposed a cross-entropy combinatorial optimization method to detect track vertical irregularity using vertical accelerations and angular velocities measured using sensors mounted on a train bogie. Quirke et al. [13] proposed a cross-entropy combinatorial optimization method that identifies railway bridge damage using bogie vertical acceleration signals from an operational train. Wei et al. [14] proposed an algorithm that distinguishes the characteristic frequencies of vertical and lateral acceleration signals of the axle box to detect crossing degradation. Wei et al. [15] proposed a mathematical model that analyzes the frequency responses of vertical and lateral acceleration signals to find track local irregularities, using bogie and car body acceleration measurements. Molodova et al. [16,17] proposed an algorithm that extracts the frequency characteristics of vertical and lateral acceleration signals and identifies insulated rail joint damage using axle box acceleration measurements. Molodova et al. [18,19] proposed an automatic method that identifies railway surface defects (i.e., squats) using axle box acceleration measurements. Molodova et al. [20] employed a three-dimensional finite element model to establish a quantitative relation between local track defects and axle box acceleration measurements to identify local isolated short-track defects. Salvador et al. [21] proposed an approach for extracting time–frequency representations of axle box acceleration signals to identify track defects, such as welded and glued joints, squats, and turnout frogs. Lee et al. [22] proposed a mixed filtering approach for finding track vertical and lateral irregularities, using axle box and bogie acceleration signals from in-service high-speed trains. This approach employs Kalman filters, bandpass filters, and compensation filters. Vinberg et al. [23] proposed monitoring methods based on quantitative signal analysis to detect the rail vehicle suspension condition and track condition, using the data on the body of a rail vehicle. Tsunashima [24] proposed a classifier based on machine learning to detect track irregularities, such as the longitudinal level, alignment, and cross level, by using measured vertical acceleration data, lateral acceleration data, and the roll rate data of the car body.

We propose a data-driven bias correction and defect diagnosis model for in-service vehicle acceleration measurements (DBCDD-IVAM). The proposed model was developed by exploring the relationship between substantial amounts of historical vibration data for the cabin of a train, as measured by track quality instruments and multiple track condition measurements from other inspection methods. The proposed method eliminates false positive detections of track vibration defects by track quality instruments and diagnoses the causes of track vibration defects (i.e., severe vertical and lateral vibrations of the cabin). 

The remainder of this paper is organized as follows. In Section 2, we present the bias in train cabin vibration measurements. The probable causes for the vibration of a train cabin are explained in Section 3, and the proposed data-driven bias correction and defect diagnosis model for in-service vehicle acceleration measurements is presented in Section 4. In Section 5, we present information on the validation of the model, using actual inspection data for the Lanxin Railway. Our conclusions are presented in Section 6.

## 2. Bias of Train Cabin Vibration Measurements

The random biases of train cabin vibration measurements are classified mainly into the following two types: (1) Mileage bias, i.e., the measured mileage of the sample point differs from the actual mileage. (2) Measurement bias, i.e., the measured values of the vertical and lateral vibration of train vehicles at the sample point differ from the real values. The main causes of these two types of bias are the rolling stock itself, vehicle models, seasonal factors, climate factors, speed, and inspection equipment or instruments.

**Figure 2:**
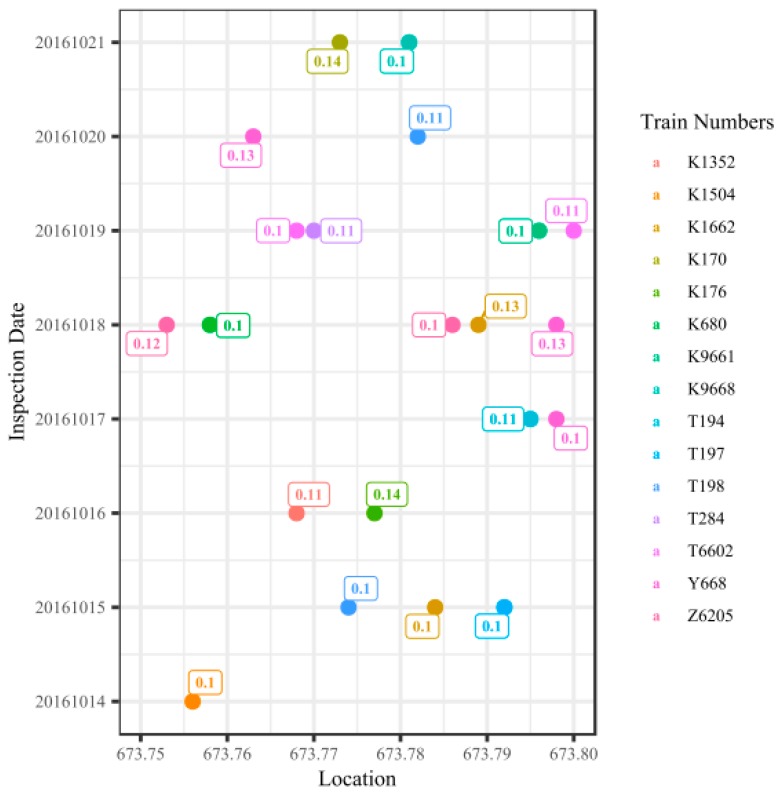
Example map of train cabin vibration measurement bias in the Lanxin Railway.

Figure 2 presents the measured values and mileages of the lateral vibration from cabin-mounted track quality instruments collected between 14 October 2016 and 21 October 2016 in the up direction of Lanxin Railway. All these measurements were caused by the same track defect, which is classified as defect type alignment and defect rank B. The defect was located between sleeper No. 54 and sleeper No. 63 of turnout No. 4 at Shangqinghe Station. Note that “up direction” means the train is bound for Beijing, the center of the Chinese railway network. The horizontal axis of the figure represents the location along the railway line (in kilometers), and the vertical axis represents the date of inspection of the track vibration defects. The differently colored dots represent different train numbers, and the numbers in the square label boxes represent the measured values of lateral vibration.

Figure 2 shows that (1) the measured values and mileages of the lateral vibration for the same track defect differ, which results from the cabin-mounted track quality instruments being fixed to different trains. The differences indicate that mileage bias and measurement bias exist in the vibration data. Therefore, based on the mileage information of the train cabin vibration defect point, it would be difficult for maintenance workers to determine the corresponding track irregularity defect. This implies a further workload on the maintenance workers, while a significant amount of work is already required from them to check and review the track vibration defects at the railway site. 

(2) Cabin-mounted track quality instruments run over the same track repeatedly, with the same track defect being detected and recorded repeatedly. However, because of the measurement bias, managers can easily mistake the same track defect for different defects. The repeated calculation arises when the track irregularity condition is evaluated. As a result, railway managers cannot determine the health condition accurately and are unable to reasonably optimize the allocation of maintenance resources.

## 3. Causes of Train Cabin Vibration

The vertical and lateral vibration measurements of train cabins can be quite substantial, indicating that the cabin vibrations in the lateral and vertical directions are severe and that the track condition is poor. Track vibration defects are defined as measured vertical and lateral vibrations of the train cabin that exceed the set thresholds. Chinese railway track maintenance rules [5,25,26,27] define the thresholds for the vibration of a train cabin in the lateral and vertical directions. The thresholds for various defect ranks for the vibration of a train cabin in the lateral and vertical directions, obtained by cabin-mounted track quality instruments (at train speeds ≤250 km/h), are shown in Table 1. The thresholds of the vertical and lateral vibrations of a train cabin obtained by portable track quality instruments (for train speeds ≤160 km/h) are shown in Table 2. Based on the thresholds, four defect ranks for vertical and lateral vibrations of the cabin are defined, namely ranks I–IV. The higher the defect rank is the more severe is the track vibration defect.

The vibration of the train cabin is affected by track irregularities and the rolling stock itself. The probable causes of severe vertical and lateral vibration of the cabin are as follows.

(1) The characteristics of the rolling stock (such as poor condition of rolling stock) or abnormal manipulation can result in the severe vertical and lateral vibration of the cabin. As—in such instances—the severe vertical and lateral vibration does not result from track irregularities, identifications of corresponding track vibration defects are false positives. Therefore, such track vibration defect data should be excluded from consideration when track health is being evaluated.

(2) Track irregularities are the main causes of train cabin vibration [28,29,30]. Track irregularities and the dynamic response of rolling stock are closely related [31]. The influences of different types of track irregularities on cabin vibration are summarized in Table 3. We can conclude that different types of track irregularities can result in similar vibrations of the train cabin. Consequently, it is not possible to identify the type of track irregularity defect based on the type of vibration of the train cabin. Note that track irregularity defects are also termed “track geometry defects”.

## 4. Data-Driven Bias Correction and Defect Diagnosis Model

### 4.1. Structure of the Proposed Model

In view of the above, we proposed a data-driven bias correction and defect diagnosis model for in-service vehicle acceleration measurements (DBCDD-IVAM) to eliminate false positive detections of track vibration defects and to diagnose the causes of track vibration defects. The proposed model comprises two parts, namely a bias correction sub-model for in-service vehicle acceleration measurements (BC-IVAM), and a defect diagnosis sub-model for in-service vehicle acceleration measurements (DD-IVAM).

Sub-model BC-IVAM is employed to identify whether the rolling stock itself or track irregularities caused the severe vertical and lateral vibration of the cabin (i.e., track vibration defects). Track vibration defects resulting from the rolling stock itself are false positives that need to be excluded from consideration in track health evaluation.

Based on the results obtained by sub-model BC-IVAM, sub-model DD-IVAM identifies which type of track irregularity defect (such as a surface or cross-level defect) causes the severe vertical and lateral vibration of the train cabin.

### 4.2. Variable Denotations

The variable denotations in the DBCDD-IVAM model are listed below.
*M*Total number of types of track irregularity defects (i.e., track geometry defects), including surface, cross level, twist, and alignment defects.*m**i*^th^ track irregularity defect type, m∈(1,2,…,M).*N*Total number of track irregularity defect ranks, including ranks A, B, and C.*n**i*^th^ defect rank of track irregularities, n∈(1,2,…,N).*V*Total number of raw samples of track vibration defects.$u_{v}$*v*^th^ raw sample of track vibration defects.*U*$U=\left(u_{1},u_{2},\ldots ,u_{V}\right)$ represents a raw sample dataset of track vibration defects.*S*Total number of samples of track vibration defects that have been addressed by the BC-IVAM sub-model.$c_{s}$*s*^th^ sample of track vibration defects that have been addressed by the BC-IVAM sub-model.*C*$C=\left(c_{1},c_{2},\ldots ,c_{S}\right)$ represents a sample dataset of track vibration defects that have been addressed by the BC-IVAM sub-model.*E*Total number of samples of historical track geometry defects, of which the defect type and defect rank are known.$p_{e}$*e*^th^ sample of historical track geometry defects, of which the defect type and defect rank are known.*P*$P=\left(p_{1},p_{2},\ldots ,p_{E}\right)$ represents a sample dataset of historical track geometry defects, of which the defect type and defect rank are known. These track geometry defects are detected by track geometry cars, track geometry trolleys, manual measurements, and the like.C_FNC_FN (a constant) represents the quantity limit of the BC-IVAM sub-model and is determined based on engineering experience and expert judgment.C_FDC_FD (a constant) represents the distance limit of the BC-IVAM sub-model and is determined based on engineering experience and expert judgment.C_FTC_FT (a constant) represents the time limit of the BC-IVAM sub-model and is determined based on engineering experience and expert judgment.C_DDC_DD (a constant) represents the distance limit of the DD-IVAM sub-model and is determined based on engineering experience and expert judgment.C_DTC_DT (a constant) represents the time limit of the DD-IVAM sub-model and is determined based on engineering experience and expert judgment.Count(•)Returns the number of the variable (•).Inf(•|condition)Returns the variable (•) that satisfies the condition.$Score(p_{e}^{mn})$Returns the scores of the track defect $p_{e}^{mn}$, the defect type of which is *m* and the defect rank of which is *n*.Type(•)Returns the defect type of the variable (•).Locate(•)Returns the location of the variable (•).FDate(•)Returns the inspection date for the variable (•).Check(•)Returns the inspection method for the variable (•).ABS(•)Returns the absolute value of the variable (•).

### 4.3. BC-IVAM Sub-Model

**Figure 3:**
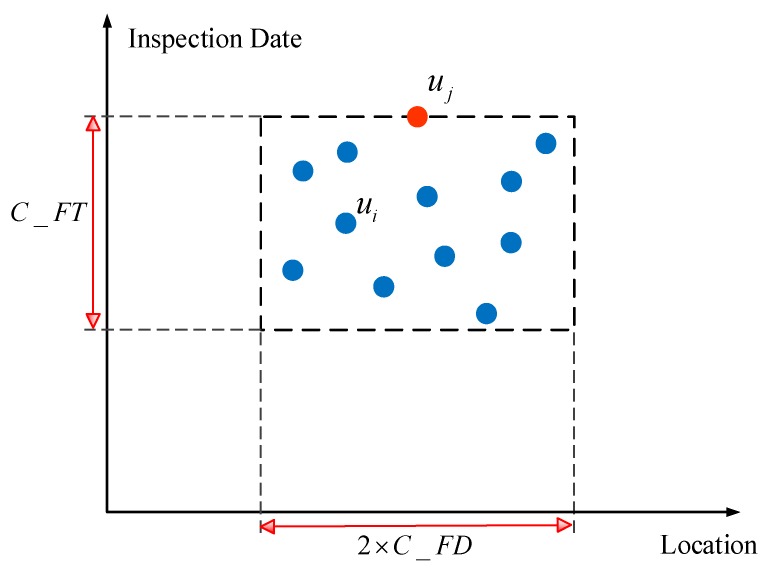
Algorithm schema of the BC-IVAM sub-model.

The inspection frequency of the track quality instruments is high, and there are substantial measurement biases in vibration data from these instruments. As a result, the same track geometry defect will be detected repeatedly by track quality instruments before the defect is repaired. The corresponding measurement points will fall within a consistent region. The algorithm schema of the BC-IVAM sub-model is shown in Figure 3. The horizontal axis represents the location of track vibration defects along the railway line, and the vertical axis represents the inspection date for track vibration defects. The points represent different track vibration defects detected at different dates. The length of the rectangular area in Figure 3 is 2×C_FD, and the width of the rectangular area is C_FT. If track vibration defect points fall within this rectangular area, these defect points are assumed to result from the same track geometry defect. If a track vibration defect point, $u_{i}$, satisfies Equations (3) and (4) the point $u_{i}$ falls within the rectangular area. In Figure 3, for example, the points $u_{i}$ and $u_{j}$ correspond to the same track geometry defect.

If the total number of track vibration defect points that fall within the rectangular area $Count\left(u_{i}\right)$ is greater than or equal to C_FN, the track vibration defect points that fall within the rectangular area are assumed to result from the same track geometry defect. These track vibration defects are not false positives. A track vibration defect point $u_{j}$ in the rectangular area that has been detected recently is selected as the representative track vibration defect, $c_{s}=u_{j}$, as shown in Equation (1). 

If the total number of track vibration defect points that fall within the rectangular area $Count\left(u_{i}\right)$ is less than C_FN, the track vibration defect points that fall within the rectangular area are assumed to result from the rolling stock itself or from the external environment. These track vibration defects are false positives.

The mathematical description of the BC-IVAM sub-model is given in Equations (1)–(4), wherein i∈[1,2,…,V], j∈[1,2,…,V], i≠j, and s∈[1,2,…,S].

(1) $$c_{s}=Inf\left(u_{j}|Count\left(u_{i}\right)\geq C\_FN,i\in \left[1,2,\ldots ,V\right],j\in \left[1,2,\ldots ,V\right],i\neq j\right)$$

where

(2) $$ABS(Locate(u_{j})-Locate(u_{i}))\leq C\_FD$$

(3) $$FDate(u_{j})-FDate(u_{i})\leq C\_FT,$$

(4) $$Check\left(u_{j}\right)=Check\left(u_{i}\right),$$

Equation (2) describes the distance constraint between the track vibration defect points. Substantial measurement biases occur in vibration measurements from track quality instruments, and the measured and actual locations of track vibration defects could differ. A single track geometry defect that has been detected repeatedly could be easily mistaken for a different track geometry defect. Therefore, the determination of whether apparently different track vibration defects are, in fact, the same defect should be based not on the kilometer point but on the kilometer range. *C_FD* is determined based on engineering experience and expert judgment.

Equation (3) describes the time constraint between track vibration defect points. If the time interval between inspection dates for two track vibration defects is too long, maintenance activities can occur during the time interval. A track geometry defect resulting in a track vibration defect that has been detected previously could have been repaired, and track geometry defects resulting in two track vibration defects could be different. The constant *C_FT* is determined based on engineering experience and expert judgment. Equation (4) expresses that the inspection method for track vibration defects is the same.

### 4.4. DD-IVAM Sub-Model

Using historical data on track geometry defects, the locations of which are close to the locations of track vibration defects, the total score for each type of track geometry defect during a certain time interval is calculated. The defect type with the largest score is selected as the most probable cause of the severe vibration of the cabin (i.e., the track vibration defect). The reasons for such selection are as follows.

Track degradation is characterized generally by memorability [30,32]. Track segments located in the same locations along the railway line have similar degradation rules after each maintenance activity. This implies that the type of track geometry defect that has been repaired before at the same location will occur repeatedly in the future. 

As there are several inspection methods for track irregularities, the same track geometry defect can be detected repeatedly before it is repaired. This implies that, based on the measurement data of the different inspection methods, the same track degradation can be described differently.

**Figure 4:**
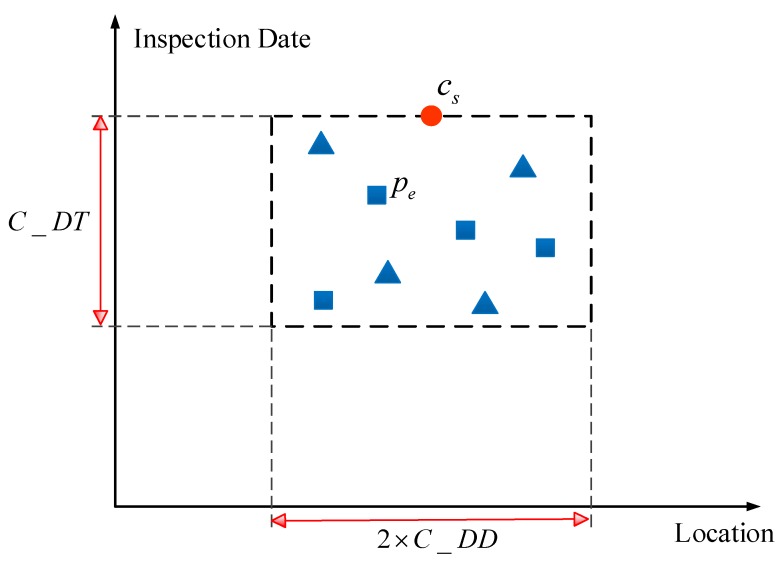
Algorithm schema of the DD-IVAM sub-model.

The algorithm schema of the DD-IVAM sub-model is shown in Figure 4. The horizontal axis represents the location along the railway line, and the vertical axis represents the inspection date of track defects. The points represent the different track defect points that are detected on different dates. The points of different shapes represent different defect types. The length and width of the rectangular area in Figure 4 are 2×C_DD and C_DT, respectively. If track geometry defect points fall within the rectangular area, these defect points are assumed to be the causes of the severe cabin vibration. In Figure 4, for example, if a track geometry defect point $p_{e}$ satisfies Equations (7) and (8), the point $p_{e}$ falls within the rectangular area and represents possible causes of the track vibration defect point $c_{s}$.

The mathematical description of the DD-IVAM sub-model is presented in Equations (5)–(8). $Type(c_{s})$ represents the defect type of the track geometry defect resulting in track vibration defect point $c_{s}$. The defect type with the largest score for track geometry defects that fall within the rectangular area is selected as $Type(c_{s})$, as shown in Equation (5), in which $p_{e}^{mn}$ represents the track geometry defect point, of which *m* is the defect type and of which *n* is the defect rank. $Score(p_{e}^{mn})$ represents the score of track geometry defect point $p_{e}^{mn}$, and it is calculated as a function of the defect rank, the inspection date, and the inspection method—as shown in Equation (6). $FDate(p_{e})$ represents the inspection date for $p_{e}$. $FDate(c_{s})$ represents the inspection date for $c_{s}$. The higher the defect rank is the closer $FDate(c_{s})$ is to $FDate(p_{e})$, the more reliable the inspection method $Check(p_{e})$ is the larger is the $Score(p_{e}^{mn})$:

(5) $$Type(c_{s})=\underset{m\in (1,2,\cdots M)}{\arg\max}\sum_{e=1}^{E}\sum_{n=1}^{N}Score(p_{e}^{mn})$$

(6) $$Score(p_{e}^{mn})=h\left(m,FDate(c_{s})-FDate(p_{e}),Check(p_{e})\right)$$

where

(7) $$ABS(Locate(c_{s})-Locate(p_{e}))<=C\_DD$$

(8) $$FDate(c_{s})-FDate(p_{e})<=C\_DT$$

Equation (7) describes the distance constraint between track vibration defect points and track geometry defect points. Equation (8) describes the time constraint between track vibration defect points and track geometry defect points. The values of the constant *C_DD* and the constant *C_DT* are determined based on engineering experience and expert judgment.

## 5. Empirical Analysis

### 5.1. Overview

In the Lanxin Railway, the inspection frequency achievable with the portable track quality instruments is once per day, whereas the inspection frequency achievable with cabin-mounted track quality instruments is three to five times per day. The vibration measurement data obtained comprise nine items: namely the line name, line direction, mileage, inspection date, defect types, defect ranks, vibration peak value, type of locomotive, and train number. The thresholds for cabin-mounted track quality instruments are shown in Table 1, and the thresholds for portable track quality instruments are shown in Table 2. 

A total of 29,176 measurements of vertical and lateral vibration of the train cabin was collected between 1 May 2016 and 30 September 2016 on the segment in the down direction of the Lanxin Railway between km 548 and km 985.5. Note that “down direction” means that the train departs from Beijing. A total of 117,408 track geometry defect measurements was collected between 1 October 2015 and 30 September 2016 over the same track segment. These defect measurements were obtained using track geometry cars, track geometry trolleys, track quality instruments, and the like. The DBCDD-IVAM model was verified using these accumulated data. The DBCDD-IVAM model was implemented using the Oracle database programming language PL/SQ [33].

**Figure 5:**
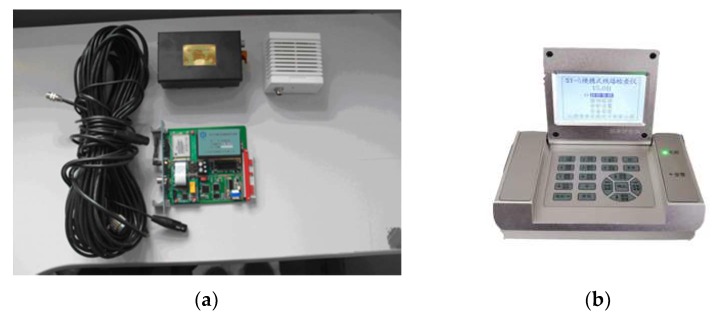
(**a**) Cabin-mounted track quality instruments (CGDJ-III) used in the Lanxin Railway; (**b**) portable track quality instruments (SY-5) used in the Lanxin Railway.

The measurement data for the vibration of the train cabin comprise two parts, namely (1) 25,942 track vibration defect data points, of which the defect rank is II or III, detected by cabin-mounted track quality instruments, as shown in Figure 5a; and (2) 3234 track vibration defect data points, of which the defect rank is III or IV, detected by portable track quality instruments, as shown in Figure 5b. These track vibration defects have been checked and reviewed by maintenance workers at the railway site, and the causes of the track vibration defects are known. 

**Figure 6:**
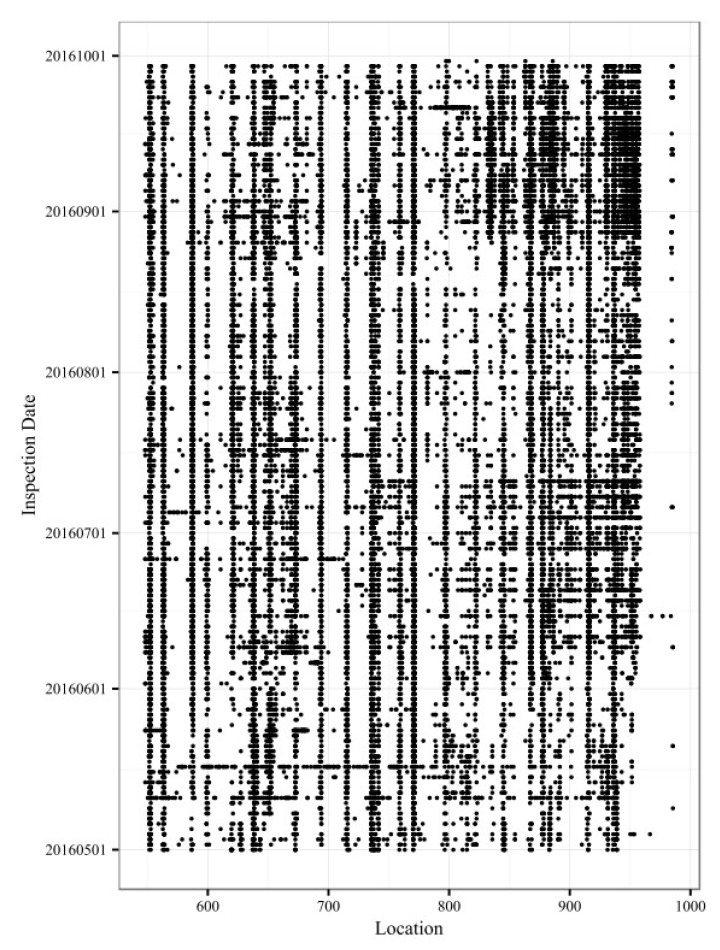
Distribution map of the track vibration defect data in the down direction of the Lanxin Railway.

The distribution map for these track vibration defect data is shown in Figure 6. The horizontal axis represents the location along the railway line (in kilometers), and the vertical axis represents the inspection date of the track vibration defects. The points represent different track vibration defect points obtained by using track quality instruments. Because the inspection frequency for track quality instruments is several times per day and because of the measurement bias, the same track geometry defect will correspond to multiple vibration defect points before the track geometry defect is repaired. Although the track vibration defect points in Figure 6 are intensively and continuously distributed in the time dimension, the actual true positive vibration defect points could be fewer.

### 5.2. Analysis of Results

#### 5.2.1. BC-IVAM Sub-Model

In the empirical analysis, the distance limit between the track vibration defect points C_FD = 25 m. The time limit C_FT = 7 days. The quantity limit for cabin-mounted track quality instruments $C\_FN_{C}$ = 5. The quantity limit for portable track quality instruments $C\_FN_{p}=3$.

The BC-IVAM sub-model provided 1898 track vibration defect points, of which 1596 were true positives. The success rate of the BC-IVAM sub-model was therefore 84.1%. Substantial bias in track vibration defect data is present in the Chinese railway management system owing to a lack of corrective processing and in-depth analysis. Therefore, Chinese railway managers can only use these vibration defect data as a reference for scheduling maintenance activities. However, the analysis performance of the BC-IVAM model improves the reliability of these vibration defect data and enhances their data value.

**Figure 7:**
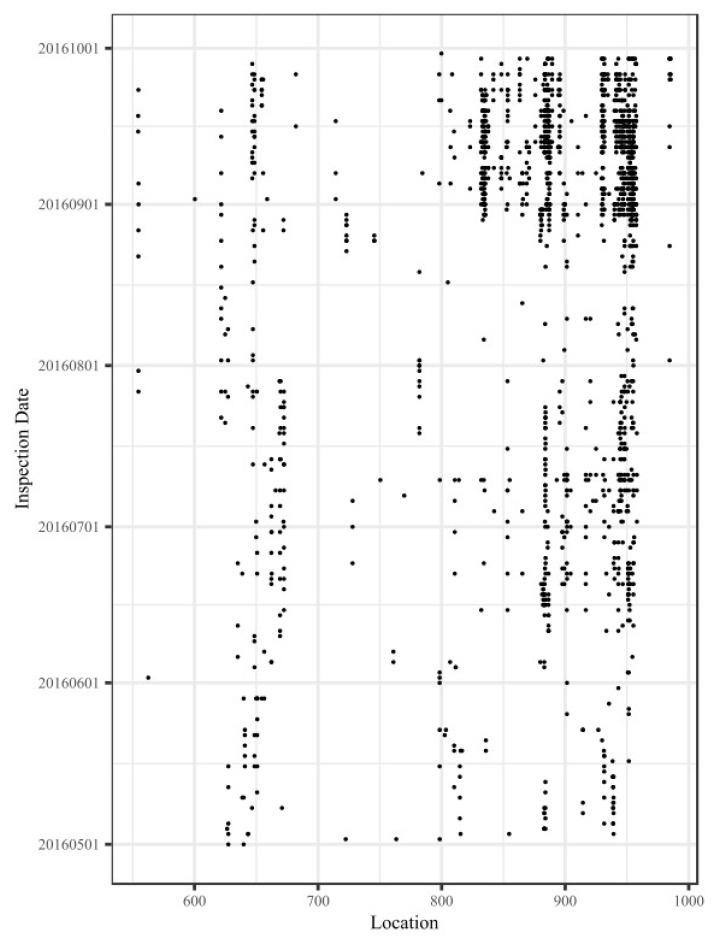
Distribution map of true positive vibration defect data in the down direction of the Lanxin Railway.

The distribution map of the corresponding true positive vibration defect data in the down direction of the Lanxin Railway is shown in Figure 7. The horizontal axis represents the location along the railway line (in kilometers), and the vertical axis represents the inspection date of the track vibration defects. Comparing Figure 6 and Figure 7 shows that the BC-IVAM sub-model performs well in obtaining the true positive vibration defect points and improving the usability of the vibration measurements.

We collected 2162 track geometry defects between 1 May 2016 and 30 September 2016 on the segment in the down direction of the Lanxin Railway between 548 km and 985.5 km. These defect measurements were obtained by using track geometry cars, track geometry trolleys, portable track quality instruments, cabin-mounted track quality instruments, and the like. The false negative rate is 26.2% for the track quality instruments, implying that 26.2% of track geometry defects are found by using the track geometry cars and track geometry trolleys. The result indicates that most track geometry defects are detected by the track quality instruments; however, it is necessary to use the track geometry cars and track geometry trolleys for periodic track inspection.

The above results indicate that the BC-IVAM sub-model performs well in identifying whether the rolling stock itself or track irregularities cause the severe vertical and lateral vibration of the cabin (i.e., track vibration defects). The model significantly improves the quality of the data on the track vibration defects. By using the BC-IVAM sub-model, the workload of maintenance workers who have to go to the railway site to check and review track vibration defects can be greatly reduced. Furthermore, unnecessary work associated with false positive detections of track vibration defects can be avoided. In addition, repetition in the records of track vibration defects can be reduced significantly, which helps to improve the accuracy of evaluating track health and track condition.

#### 5.2.2. DD-IVAM Sub-Model

In the empirical analysis, the distance limit between track vibration defect points and track geometry defect points C_DD = 25 m. The time limit C_DT = 30 days. The scores of the track geometry defect point $p_{e}^{mn}$, $Score(p_{e}^{mn})$ are calculated as shown in Equation (9):

(9) $$Score(p_{e}^{mn})\left\{ \begin{array}{l} 5,\begin{array}{cc} & n=\prime A\prime \end{array}\\ 2,\begin{array}{cc} & n=\prime B\prime \end{array}\\ 1,\begin{array}{cc} & n=\prime C\prime \end{array}\\ 0.5,\begin{array}{cc} & else \end{array} \end{array}\right.$$

A total of 1209 track vibration defect points was diagnosed correctly. The success rate of the DD-IVAM sub-model was 75.8%. Currently, considerable time and effort are being expended by maintenance workers to check and review track vibration defects. Our results indicate that the DD-IVAM sub-model performs well in track vibration defect diagnosis, and reduces the workload associated with track checking and reviewing. In future, the results of the defect diagnosis can be used as a valid basis for managers to arrange maintenance activities.

The confusion matrix employed to compare the diagnosed and actual causes of track vibration defects is shown in Table 4. The elements in the matrix represent the number of defect points, where the actual causes are listed as rows and the diagnosed causes are listed as columns. The main diagonal elements represent the number of correctly diagnosed defect points.

**Figure 8:**
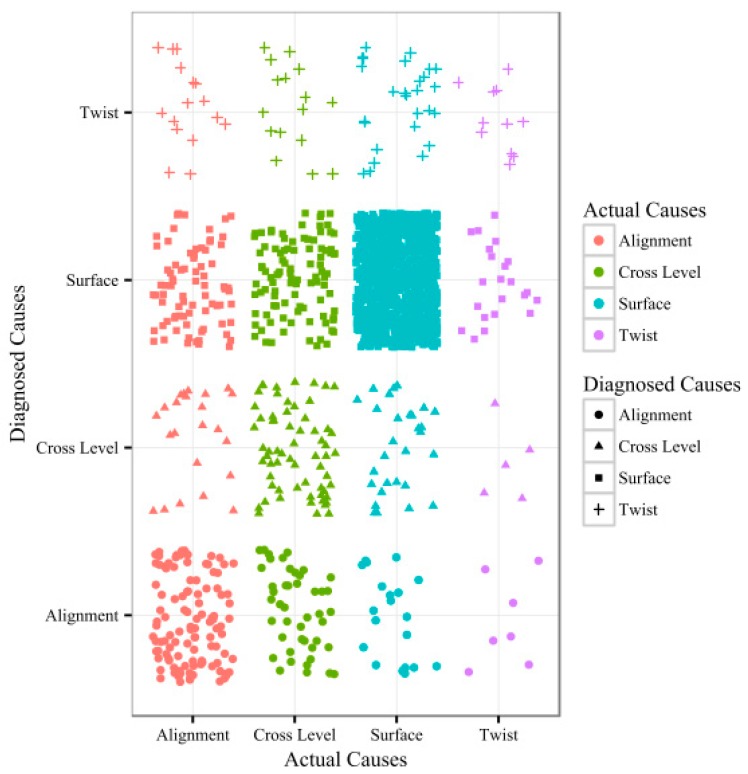
Scatter plot of the diagnosed and actual causes of track vibration defects.

A scatter plot of the diagnosed and actual causes of track vibration defects is shown in Figure 8. The horizontal axis represents the actual causes and the vertical axis represents the diagnosed causes. Points in different colors represent the defect points that have different actual causes. Points in different shapes represent the defect points that have different diagnosed causes.

The following conclusions can be drawn from the results presented in Figure 8 and Table 4. The main diagonal elements are large numbers, and the majority of causes of track vibration defects are correctly diagnosed. These findings indicate that the DD-IVAM model can accurately diagnose the causes of track vibration defects. 

Using the DD-IVAM sub-model, the appropriateness of the work tasks that railway staff perform to check and review track vibration defects can be improved, and track maintenance-window utilization can be increased. In addition, using in-service vehicle acceleration measurements, the DD-IVAM sub-model can be employed to help managers become aware of near “real-time” track conditions.

## 6. Conclusions

In this study, we proposed a data-driven bias correction and defect diagnosis model for in-service vehicle acceleration measurements (DBCDD-IVAM) based on the degradation characteristics of railway tracks. The proposed model comprises two parts, namely a bias correction sub-model for in-service vehicle acceleration measurements (BC-IVAM) and a defect diagnosis sub-model for in-service vehicle acceleration measurements (DD-IVAM). The BC-IVAM sub-model is used to eliminate false positive track vibration defects detected by track quality instruments, and the DD-IVAM sub-model is used to diagnose the causes of the severe vertical and lateral vibration of the cabin (i.e., track vibration defects). The probable causes of track vibration defects include surface, cross level, twist, and alignment defects. The proposed model helps to increase the value of the vibration data obtained from track quality instruments and helps track maintenance workers and managers to evaluate track health accurately and assess track condition trends—both of which are crucial for optimal scheduling of maintenance and repair events.

The proposed model was verified using actual data collected between 548 km and 985.5 km in the down direction along the Lanxin Railway. The success rate of the BC-IVAM sub-model was 84.1% and that of the DD-IVAM sub-model 75.8%. These results show that the BC-IVAM sub-model performs well in identifying false positive track vibration defects and the DD-IVAM sub-model performs well in track vibration defect diagnosis.

We intend to conduct further research, to follow the directions mentioned below.

(1) To further validate and improve the DBCDD-IVAM model, more track vibration defect data with known causes need to be collected. 

(2) To improve the accuracy of the model in identifying defect types, we intend studying the use of gyro sensor data.

(3) Using the track geometry wave data from track inspection cars, based on the similarity principle, we intend to investigate the location error correction of measurement data from track quality instruments in future studies. 

(4) We intend to combine the axle box acceleration data and gyro sensor data with other data sources to identify the track geometry defect rank. Such information can assist managers in arranging maintenance activities based on scientific analyses.

(5) Furthermore, we intend on using the DBCDD-IVAM model in an attempt to upgrade and improve the measurement accuracy of the track quality instruments.

## Figures and Tables

**Table 1:** Thresholds of vertical and lateral vibrations of the cabin of a train obtained by cabin-mounted track quality instruments (for train speeds ≤250 km/h).

Type of Train Cabin Vibration	Rank I	Rank II	Rank III	Rank IV
Vertical train cabin vibration (g)	0.10	0.15	0.20	0.25
Lateral train cabin vibration (g)	0.06	0.10	0.15	0.20

**Table 2:** Thresholds of vertical and lateral vibrations of a train cabin obtained by portable track quality instruments (for train speeds ≤160 km/h).

Type of Locomotive	Type of Train Cabin Vibration	Rank I	Rank II	Rank III	Rank IV
Shaoshan 9	Vertical train cabin vibration (g)	0.12	0.15	0.24	0.45
	Lateral train cabin vibration (g)	0.08	0.10	0.22	0.40

**Table 3:** Influence of different types of track irregularities on train cabin vibration.

Track Irregularity Defect Type	Vibration of Train Cabin
Surface	Large vertical vibration
Cross level	Large vertical vibration
	Large lateral vibration
Twist	Large vertical vibration
	Large lateral vibration
Alignment	Large lateral vibration
Gauge	-

**Table 4:** Confusion matrix for diagnosed and actual causes of track vibration defects.

		Diagnosed Causes (i.e., Track Irregularity Defect Types)				Sum
		Alignment	Cross level	Surface	Twist	
Actual causes (i.e., track irregularity defect types)	Alignment	111	23	78	16	228
	Cross level	45	64	99	16	224
	Surface	20	29	1023	27	1099
	Twist	7	5	22	11	45
Sum		183	121	1222	70	1596
